# Blocking spinal CCR2 with AZ889 reversed hyperalgesia in a model of neuropathic pain

**DOI:** 10.1186/1744-8069-6-90

**Published:** 2010-12-10

**Authors:** Alexandre Serrano, Michel Paré, Fraser McIntosh, Steven JR Elmes, Giovanni Martino, Claudia Jomphe, Etienne Lessard, Paola MC Lembo, François Vaillancourt, Martin N Perkins, Chang Qing Cao

**Affiliations:** 1 AstraZeneca R&D Montréal, 7171 Frédérick Banting, Ville St-Laurent (Montréal) Québec, Canada, H4S 1Z9; 2 Pharsight, a Certara™ Company, 2000 Peel Street, Suite 570, Montreal, Quebec, Canada, H3A 2W5

## Abstract

**Background:**

The CCR2/CCL2 system has been identified as a regulator in the pathogenesis of neuropathy-induced pain. However, CCR2 target validation in analgesia and the mechanism underlying antinociception produced by CCR2 antagonists remains poorly understood. In this study, *in vitro *and *in vivo *pharmacological approaches using a novel CCR2 antagonist, AZ889, strengthened the hypothesis of a CCR2 contribution to neuropathic pain and provided confidence over the possibilities to treat neuropathic pain with CCR2 antagonists.

**Results:**

We provided evidence that dorsal root ganglia (DRG) cells harvested from CCI animals responded to stimulation by CCL2 with a concentration-dependent calcium rise involving PLC-dependent internal stores. This response was associated with an increase in evoked neuronal action potentials suggesting these cells were sensitive to CCR2 signalling. Importantly, treatment with AZ889 abolished CCL2-evoked excitation confirming that this activity is CCR2-mediated. Neuronal and non-neuronal cells in the spinal cord were also excited by CCL2 applications indicating an important role of spinal CCR2 in neuropathic pain. We next showed that in vivo spinal intrathecal injection of AZ889 produced dose-dependent analgesia in CCI rats. Additionally, application of AZ889 to the exposed spinal cord inhibited evoked neuronal activity and confirmed that CCR2-mediated analgesia involved predominantly the spinal cord. Furthermore, AZ889 abolished NMDA-dependent wind-up of spinal withdrawal reflex pathway in neuropathic animals giving insight into the spinal mechanism underlying the analgesic properties of AZ889.

**Conclusions:**

Overall, this study strengthens the important role of CCR2 in neuropathic pain and highlights feasibility that interfering on this mechanism at the spinal level with a selective antagonist can provide new analgesia opportunities.

## Background

Neuropathic pain treatment is often refractory to available therapies and its underlying mechanisms remain poorly understood. This pathological state reflects abnormal sensory processes caused by a variety of cellular changes that result in abnormal hyperexcitability, hyperactivity and spontaneous activity in the pain circuitry [1]. Many molecular events have been implicated for their contribution to neuropathic pain. The CC chemokine ligand 2 (CCL2 or monocyte chemoattractant protein-1/MCP-1) and its CC chemokine receptor (CCR2) are of interest as they have recently been shown to be overexpressed in glial and neuronal cells following injury to the nervous system and may contribute to the neuroinflammatory processes associated with the development and maintenance of neuropathic pain [2-14]. In addition, mice lacking CCR2 receptors failed to show mechanical allodynia in the partial nerve injury model [2] while antagonists of CCR2 reversed nociceptive responses in the spinal nerve ligation and varicella zoster animal model [15] as well as in the focal demyelination of the sciatic nerve model of neuropathic pain [4].

Although a growing body of evidence suggests that interventions aiming to block CCR2/CCL2 signalling may alleviate neuropathic pain, little is known about the actual cellular site of action of this effect. So far, there appears to be a disagreement on the site of action of CCR2 antagonists producing analgesia since studies have provided evidence that peripheral and central nervous system (CNS) mechanisms may be involved. Some studies have suggested that both resident and infiltrating spinal microglia activated by CCR2 [14] contributed to enhanced neuronal excitation [16] during the development of nerve injury induced neuropathy. Others suggested that CCR2 antagonists can inhibit activation of the sciatic nerve and DRG neurons which supports a peripherally-mediated analgesia mechanism [17]. Finally, DRG neurons activated by CCL2 could perhaps contribute to both centrally- and peripherally-mediated pathophysiology [18-20].

Here we provide further details on the cellular and pharmacological mechanisms of CCL2/CCR2 signalling in a model of neuropathic pain through the integration of cellular imaging, electrophysiology as well as the use of AZ889, a competitive CCR2 blocker. In addition, the behavioural evaluation of AZ889 in the CCI model of neuropathic pain was supplemented via critical pharmacokinetic measures of drug exposure that strengthened the *in vitro *to *in vivo *translation of pharmacological properties.

## Results

### Identification of the potent CCR2 antagonist

**Figure 1:**
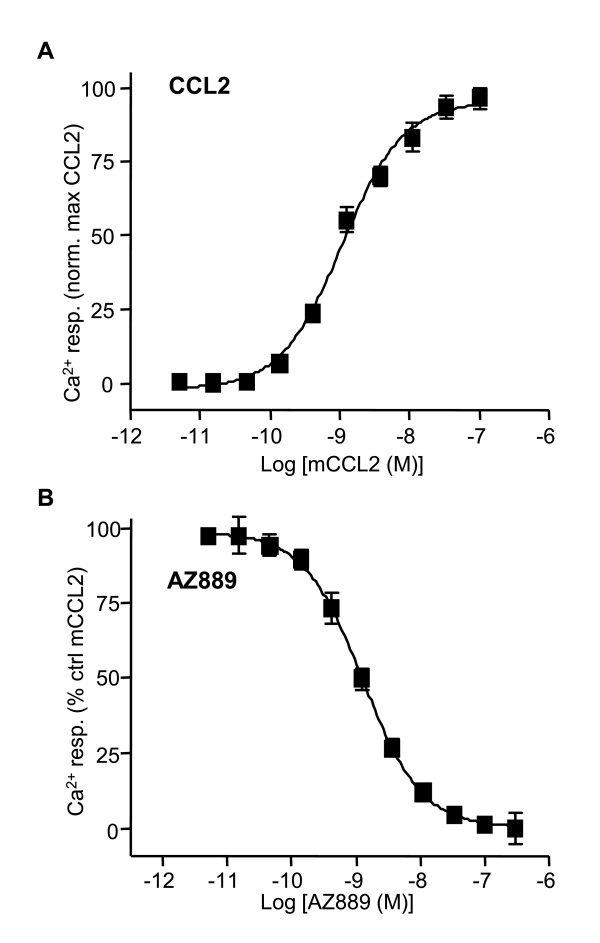
**mCCL2-induced intracellular calcium mobilization in HEK293 s cells expressing CCR2 is blocked by compound AZ889**. (A) Mouse derivative of CCL2 evoked calcium activation of HEK293 s cells expressing CCR2 with an EC50 value of 1 nM. Calcium mobilization was recorded on a FLIPR system. (B) AZ889 dose-dependently inhibited CCR2-mediated calcium response evoked by 1 nM of mCCL2 with an IC50 value of 1.3 ± 0.2 nM (n = 5). The values were normalized to control mCCL2-evoked responses. The data shown is the average of 3 independent experiments.

Chemokine receptors are known to modulate intracellular calcium concentration [21]. A library of designed molecules was screened on HEK cells expressing the receptor using a calcium flux-assay (FLIPR). Compounds capable of blocking the intracellular calcium rise evoked by mouse CCL2 in HEK293 s cells (Gαqi5) stably expressing the rat CCR2 receptor were selected and further profiled by performing dose-response curves. Mouse and rat CCL2 (mCCL2 and rCCL2) purchased from R&D Systems gave similar median effective concentrations (EC50) values (Additional file 1 Figure S1) and mCCL2 was selected to conduct the screening assay. Cells were pre-incubated (30 min) with a given concentration of compound (from 0.04 to 1 μM) and CCR2 calcium-mediated activation was evoked by the addition of mCCL2 (EC50 concentration, 1 nM). A potent antagonist, AZ889, was identified for competitively inhibiting mCCL2-evoked calcium response with an observed median inhibition concentration (IC50) potency value of 1.3 ± 0.2 nM (n = 5; Figure 1).

The selectivity of AZ889 was further assessed using a broad panel of targets, including (G protein-coupled receptors) GPCRs, ligand-gated receptors, ion channels, transporters and enzymes. AZ889 was inactive (less than 50% effect at 10 μM) on all targets (Additional file 2 Table S1). Since the rat CCR2 receptor shares 85% homology at the amino acid level with CCR5, AZ889 was further profiled on CHO cells stably expressing rat CCR5 and stimulated with the CCR5 ligand macrophage inflammatory protein 1alpha (MIP-1α). Using the calcium-mobilization assay, AZ889 caused a concentration-dependent inhibition of MIP-1α-evoked responses with an IC50 value of 79 nM confirming AZ889 has 60-fold selectivity for CCR2 as compared to CCR5 (data not shown).

### Modulation of DRG neuron firing by CCL2

**Figure 2:**
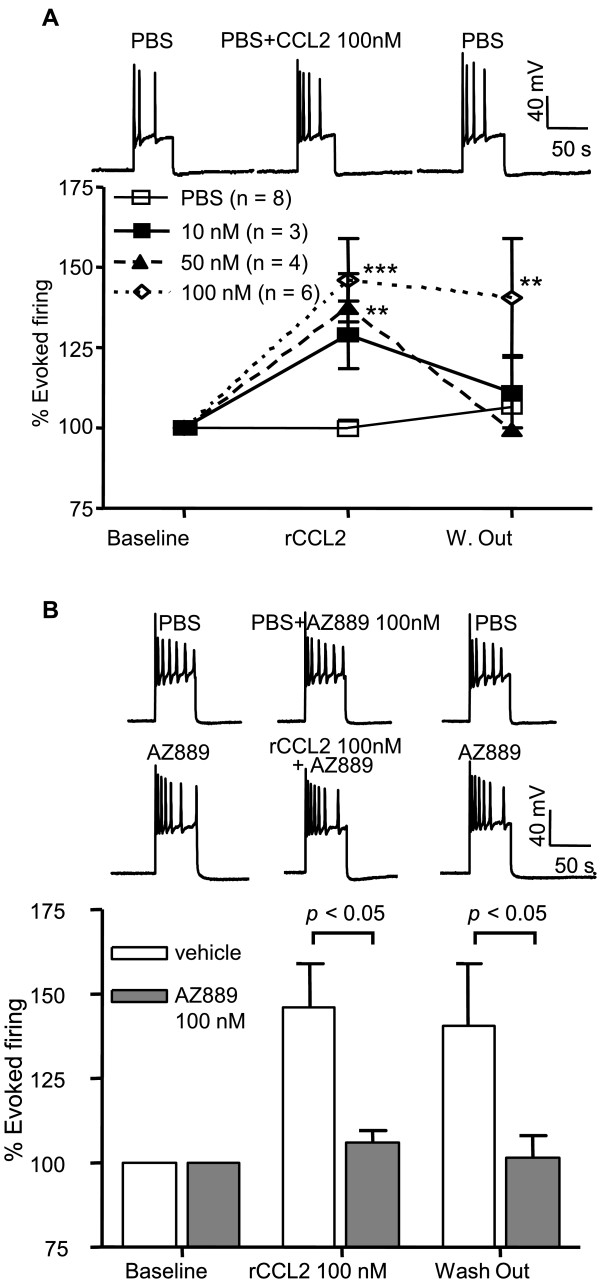
**CCL2 modulates action potential firing frequency in DRG neurons**. (A) Current-evoked action potentials from a representative DRG neuron from CCI rat before during and after application of CCL2. rCCL2 evoked a concentration-dependent increase in firing during a depolarizing current step in DRG neurons. **p < 0.01, ***p < 0.001 vs vehicle. (B) Summary data showing the efficacy of AZ889 to block rCCL2-mediated excitation of neuropathic DRG neurons. *p < 0.05 vs vehicle.

Previous studies have reported that rat DRG neurons from chronic compression of DRG (CCD) neuropathic model are activated by CCL2 applications which triggers an increase in action potentials generated in these cells [19]. To assess whether DRG neurons from rats with chronic constriction injury (CCI) were also CCL2-sensitive, we performed whole-cell patch clamp recordings to evaluate the effect of the chemokine on cell excitability. A suprathreshold (500 ms) depolarizing current step was used to evoke a stable baseline of action potentials (Figure 2A) in the absence or presence of locally applied CCL2 (1min) onto the recorded cell. CCL2 at 10 nM produced a reversible 29 ± 9% increase in evoked firing compared to vehicle (*p *< 0.01, n = 3, Figure 2A). The maximal concentration of 100 nM rCCL2 facilitated neuronal firing by 46 ± 13% (*p *< 0.01, n = 6). Application of the selective CCR2 receptor antagonist, AZ889 (100 nM), did not affect evoked neuronal firing (Figure 2B, n = 6). However, pre-treatment with AZ889 (100 nM) prior to local application of rCCL2 (100 nM) completely prevented the agonist-induced increase in firing (*p *< 0.01, n = 13; Figure 2B) confirming that rCCL2 effects were mediated by CCR2. The rCCL2-activated DRG neurons were all sensitive to transient receptor potential channel V1 (TRPV1) agonist capsaicin suggesting these cells were small nociceptive neurons (data not shown).

### CCL2 evokes calcium-mediated activation of both neuronal and non-neuronal cells from CCI rats

**Figure 3:**
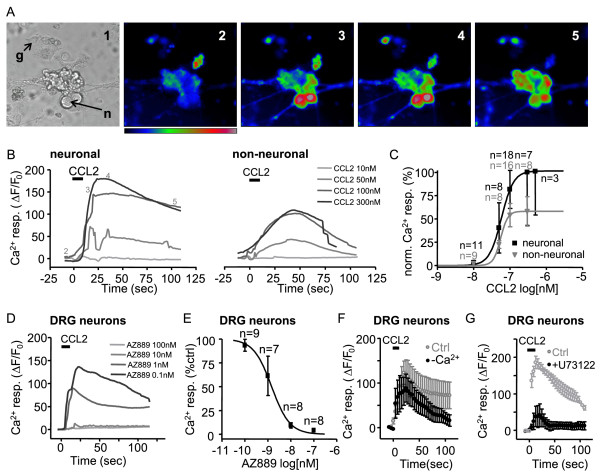
**CCL2 evokes a calcium-mediated activation of DRG neuronal and non-neuronal cells**. A) Representative neuronal (n) and non-neuronal (g) in bright field (1) and at various times of the fluorescent calcium imaging recording (2-5) indicated in B. Concentration-dependent calcium rise evoked by CCL2 (black bar, 10, 50, 100 and 300 nM) in DRG neurons (left) and in non-neuronal cells (right). Traces are average calcium responses for all recorded cells. C) Calcium rise peak normalized to maximal neuronal response represented in a concentration-response curve (EC50 58.5 nM and 55.6 nM for neuronal and non-neuronal cells respectively). D) and E) Average calcium response curves evoked by 100 nM CCL2 (black bar) in DRG neurons in the presence of AZ889 0.1 nM (n = 9 cells), 1 nM (n = 7 cells), 10 nM (8 cells) and 100 nM (8 cells) normalized to control responses without AZ889 (IC50 1.5 nM). F) Calcium rise evoked by 100 nM CCL2 (black bar) in DRG neurons from CCI rats bathed in control conditions (grey) and in the absence of extracellular calcium (black). Peak amplitudes in control and in the absence of extracellular calcium are not significantly different (p > 0.05). G) Calcium rise evoked by 100 nM CCL2 (black bar) in DRG neurons from CCI rats bathed in control conditions (grey) and in the presence of U73122 (1 μM). U73122 produced a significant block of the calcium rise peak (p < 0.05).

An increased number of action potentials in DRG neurons following CCR2 activation is an intriguing observation since CCR2 is a pertussis toxin (PTX)-sensitive GPCR coupled to inhibitory Gi and/or Gq [22,23]. Additionally, CCR2 activation evokes excitatory Ca^2+ ^rises in DRG cells [4,24] but the underlying mechanism is still poorly understood. To gain further insight into CCR2-mediated Ca^2+ ^mobilization, we next evaluated the sensitivity of both neuronal and non-neuronal cell types in the DRG by recording changes in intracellular calcium evoked by CCL2. Interestingly, CCL2 did not evoke any response in DRG cells harvested from naive rats (data not shown). In mixed-cell cultures from CCI rats, CCL2 evoked an intracellular calcium rise in both neuronal and non-neuronal DRG cells (Figure 3A-C). Non-neuronal cells, satellite cells or microglia, were easily distinguished from neurons by their size, morphology and absence of dendritic processes (Figure 3A). The amplitude of the calcium rise evoked by rCCL2 in cultured DRG cells was concentration-dependent and the calculated EC50 value was 59.6 nM in neurons and 56.7 nM in non-neuronal cells confirming both DRG cell types were CCL2-sensitive (Figure 3C).

Since rCCL2 can activate both CCR2 and CCR5 [25] we used AZ889 to test if a selective CCR2 antagonist can prevent CCL2-induced calcium signalling in native tissue. In DRG neurons, incubation with AZ889 (5 min) produced a concentration-dependent inhibition of the rCCL2 (100 nM) evoked calcium response with an IC50 of 1.5 nM (Figure 3D-E). This confirms that neuropathic DRG neurons show functional CCR2 signalling mediated by an intracellular calcium rise.

### Mechanism underlying calcium mobilization evoked by CCR2 activation in DRG neurons

To understand the intracellular mechanism involved in CCL2-mediated calcium mobilization we next tested if CCR2 activation produced a release of calcium from internal stores. In the absence of extracellular calcium, the peak CCL2-evoked calcium rise was reduced, but this difference was not significant (*p *> 0.05, n = 9 cells, Figure 3F) suggesting that intracellular stores are the principal source of calcium mediating this response. It has been previously reported that CCR2 receptors signal through phospholipase C/inositol trisphosphate (PLC/IP3) -dependent pathways to intracellular calcium stores in DRG neurons from focal demyelination mouse model [24]. To evaluate the contribution of this pathway in DRG pathophysiology, we used the PLC blocker U73122. When DRG neurons where incubated in 1 μM U73122 before (5 min), during and after CCL2 application, the evoked calcium mobilization was significantly blocked (p < 0.05, n = 6 cells, Figure 3G) suggesting that activation of the PLC/IP3 pathways is necessary for the CCR2 function in DRG neurons from CCI rats.

### CCR2 antagonism attenuates mechanical and thermal hyperalgesia in neuropathic CCI animals

**Figure 4:**
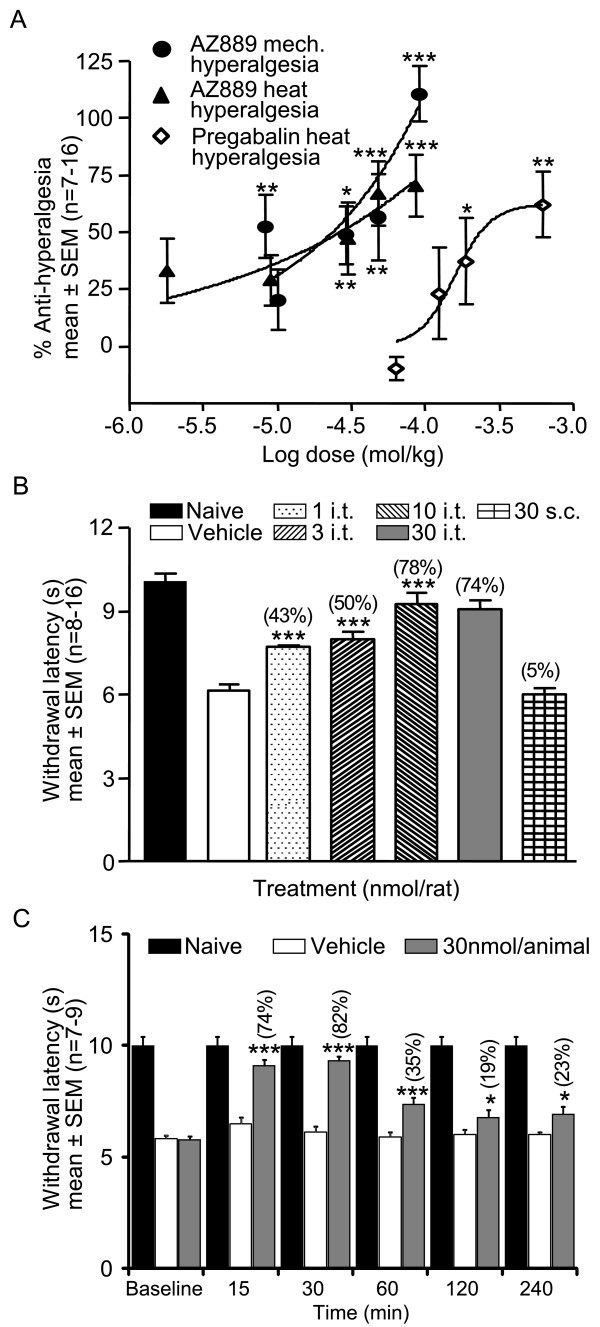
**Mechanical and heat hyperalgesia in CCI rats**. (A) Oral administration of AZ889 reverses mechanical and heat hyperalgesia in the CCI rats (tested 4 h after dosing). Oral administration of pregabalin reverses heat hyperalgesia in CCI rats (tested 1 h after dosing). (B) Intrathecal injection of AZ889 (1-30 nM/15 μL) reverses withdrawal latencies to heat stimulation of the affected paw in CCI rats (tested 15 min after dosing). Normalized values in parenthesis on top of the bar show mean % effect for each treatment. (C) Time course of the reversal of heat hyperalgesia after intrathecal injection of AZ889 (30 nmol/animal) in CCI rats (tested 15, 30, 60, 120 and 240 min after dosing). All panels, *p < 0.05, **p < 0.01, ***p < 0.001 vs vehicle (i.t. study: one-way ANOVA on the raw data and Holm-Sidak post hoc test). Normalized values in parenthesis on top of the bar show mean % effect for each time point.

The contribution of CCR2 signalling to neuropathic conditions was tested by the ability of AZ889 to reverse hyperalgesia in vivo. For this procedure, CCI rats were tested 4 h post administration, corresponding to the time of peak plasma exposure as determined by the pharmacokinetic profile (Table 1). When compared with naive animals, rats with a chronic constriction injury to the sciatic nerve showed a significant decrease in the paw withdrawal latencies to radiant heat (10.6 ± 0.4 sec in naive vs 5.9 ± 0.4 sec in CCI, *p *< 0.001, data not shown) and mechanical stimulation (146.4 ± 6.0 g in naive vs 92.5 ± 4.0 g in CCI, *p *< 0.001, data not shown). Systemic administration (p.o.) of AZ889 dose-dependently reversed mechanical and thermal hyperalgesia responses compared to vehicle-treated neuropathic animals (*p *< 0.001, Figure 4A, see Table 2 for EC50). Importantly, during behavioural studies, no overt signs of sedation as reported by the locomotor activity assay, convulsion or aggression were noted up to the maximal dose of AZ889.

**Table 1:** Pharmacokinetic profile of AZ889 with P.O. administration

Dose (μmol/kg)	Cmax (μM)	Tmax (h)	Bioavailability (%)
35.03	0.172	4	13.9

**Table 2:** Analgesic profile of AZ889 in CCI neuropathic rats

	**Plasma-EC**_**50 **_**(free**^**#**^**)**	**Brain-EC**_**50 **_**(free**^**#**^**)**	Effect at 48 μmol/kg
	**nM**	**nM**	**% (± SEM)**

Mechanical	33	3.8	58 ± 16*
Thermal	80	6.1	60 ± 12*

^# ^Plasma protein binding = 82.6%, brain protein binding = 93.9%.

* Significantly different (*p *< 0.001) from vehicle treated animals (n = 6-14)

To compare the efficacy of AZ889 with a clinically validated analgesic, a dose-response study with pregabalin was also conducted. Pregabalin (p.o.) reduced heat hyperalgesia with a potency 10-fold lower than AZ889 (Figure 4A). Importantly, a clear decrease in muscle tone was observed in animals treated with the highest dose of pregabalin (100 mg/kg p.o.) whereas no such effect was observed with AZ889 (data not shown).

### Site of action of CCR2-mediated analgesia

Pharmacokinetic data revealed that the brain to plasma ratio of AZ889 was 0.23 indicating this compound penetrates the central nervous system. Free brain concentration of AZ889 in satellite animals showed that pharmacologically relevant concentrations relative to in vitro potency and selectivity of the compound at CCR2 (within 3- to 5-fold in vitro IC50) were sufficient to observe thermal and mechanical anti-hyperalgesia effects in neuropathic animals (Table 2). Based on the close relationship between free brain concentration and the *in vitro *IC50 of AZ889, we wanted to confirm that the site of action of the analgesic effect of AZ889 was centrally mediated. Thus, spinal intrathecal administrations of low doses of AZ889 were tested for their anti-hyperalgesic abilities in rat behavioural assays. As a control, the highest dose was also given subcutaneously. Intrathecal doses of AZ889 ranging from 1 to 30 nmol/rat, produced a dose-dependent reversal of heat hyperalgesia (ED50 of 2.3 nmol; maximal reversal of hyperalgesia of 78%; Figure 4B) supporting an important central site of action. Subcutaneous administrations of the same dose of AZ889 (30 nmol/rat) showed no effect, hence ruling out the possibility that i.t. administered compound acted outside the CNS.

To determine the time course of the effect of AZ889, animals were tested 15, 30, 60, 120 and 240 minutes following intrathecal administration of 30 nmol/animal of AZ889. Time-dependent reversal of thermal hyperalgesia responses by AZ889 was compared to vehicle-treated neuropathic animals (*** p < 0.001 and * p < 0.05, Figure 4C) and naive animals. The peak reversal of hyperalgesia was observed 15-30 min post-injection.

### Mechanical- and electrical-evoked nociceptive input to the spinal cord in CCI rats involves CCR2 signalling

**Figure 5:**
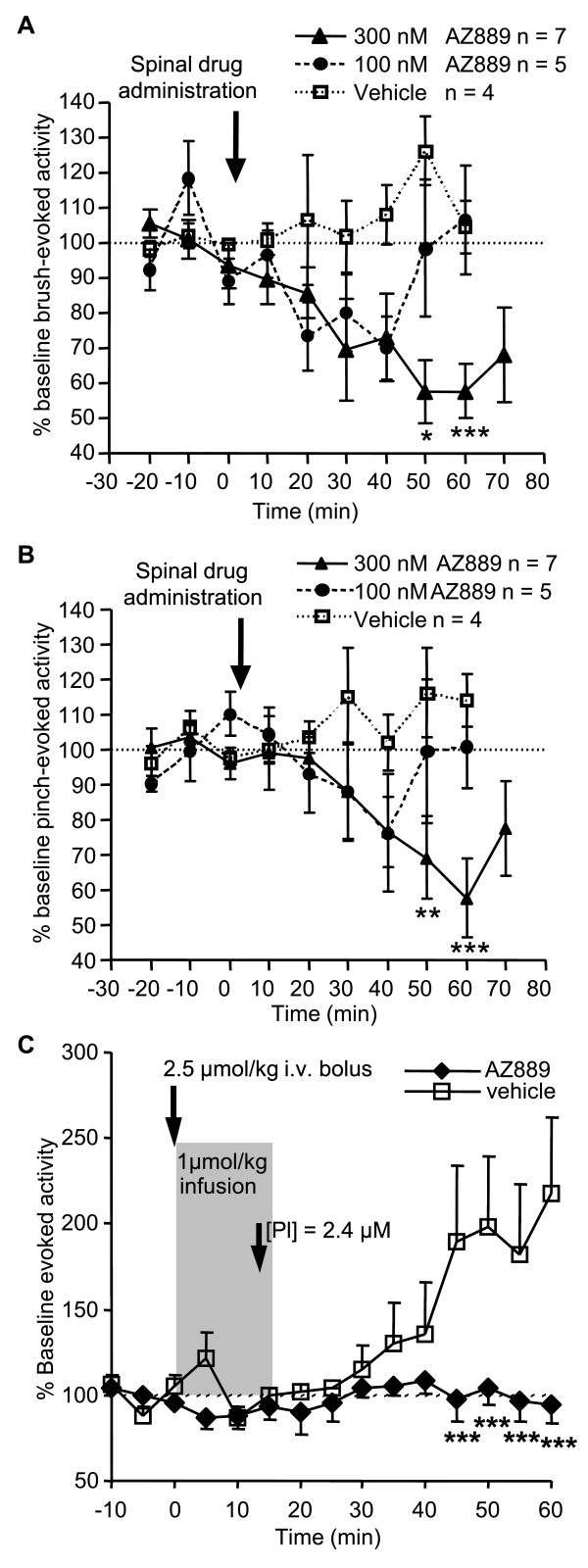
**Evoked neuronal spinal activity in CCI rats involves CCR2 signalling**. (A) Innocuous brush-evoked activity of WDR neurons in CCI rats before, during and after spinal administration of 100 nM and 300 nM AZ889. *p < 0.05, ***p < 0.001 vs vehicle (two-way ANOVA and Bonferonni post hoc test). (B) Noxious pinch-evoked WDR neuronal activity before, during and after spinal administration of 100 nM and 300 nM AZ889 in CCI rats. **p < 0.01, ***p < 0.001 vs vehicle (two-way ANOVA and Bonferonni post hoc test). (C) Flexor alpha-motoneuron response to repetitive low frequency C-afferent fibre volleys to the sural nerve in vehicle- and AZ889-treated CCI rats. AZ889 i.v. infusion of 1 μmol/kg in 15 min preceded by a loading dose of 2.5 μmol/kg (2.4 μM plasma concentration) blocked wind-up response. ***p < 0.0001 vs vehicle.

To further investigate the mode of action of spinal CCR2 antagonism, *in vivo *single cell neuronal recordings were tested on evoked neuronal activity in CCI animals. Administration of AZ889 directly to the exposed spinal cord produced a significant dose-dependent inhibition of single-unit wide dynamic range (WDR) neuron activity evoked by innocuous brush (300 nM *p *< 0.05 compared to vehicle) and noxious pinch (300 nM *p *< 0.01 compared to vehicle) suggesting an involvement of spinal CCR2 in modulating somatosensory transmission in neuropathic conditions. Maximal inhibition of WDR brush-evoked firing was sustained between 50 and 60 minutes (57.5 ± 8.9% and 57.8 ± 7.9% of baseline activity respectively) post spinal drug administration (Figure 5A). Sixty minutes after spinal drug administration, noxious pinch-evoked WDR firing decreased to 57.8 ± 11.3% of baseline activity (Figure 5B).

In addition, we evaluated if AZ889 interfered with spinal sensitization. We used repetitive electrical stimuli at noxious intensity to induce a spinally-mediated N-methyl-D-aspartic acid (NMDA)-dependent progressive hypersensitivity of nociceptive flexor reflex, e.g. wind up [26-28]. In control conditions, repeated low frequency electrical stimulation of the sural nerve ipsilateral to the injured paw of CCI rats produced sensitization of the evoked responses (Figure 5C). AZ889 significantly blocked this phenomenon (*p *< 0.0001, n = 6) suggesting this compound produced analgesia by interfering with NMDA-dependent spinal wind-up. Intravenous infusion of AZ889 (2.5 μmol/kg bolus + 1 μmol/kg/0.25 h) achieved a plasma concentration of 2.4 μM confirmed by measuring plasma concentration in a test animal.

### CCL2 activates neuronal and non-neuronal cells in spinal cord slices from CCI rats

**Figure 6:**
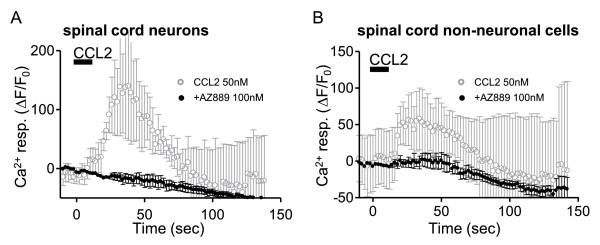
**Neuronal and non-neuronal cells in spinal cord slices from CCI rats are responsive to CCL2 applications**. Both neuronal (A, grey trace) and non-neuronal cells (B, grey trace) are responsive to 50 nM CCL2 application (black bar) in spinal cord slices from CCI rats. Average traces from 8 neuronal and 6 non-neuronal cells in 4 slices. Error bars show standard error. Pre-incubation of the spinal cord slices in AZ889 (100 nM) before (5 minutes), during and after the application of rCCL2 prevented the calcium response in both neuronal and non-neuronal cells (A and B, black traces).

A prerequisite for the involvement of the spinal cord in analgesia produced by AZ889 is that spinal cells are activated by CCL2. To evaluate this we used spinal cord slices from CCI rats and evaluated if neuronal and non-neuronal cell types were sensitive to CCL2. For these recordings, calcium imaging acquisition was focused on lamina I of the dorsal horn. Non-neuronal cells typically displayed a higher baseline fluorescence level and were distinguishable by their size and stellar morphology different from the large round shape of the neuronal cell bodies. In some experiments, neurons were recorded in patch clamp to confirm the presence of action potentials upon depolarization (data not shown). In this region, non-neuronal cells included astrocytes, microglia and other types of glial cells. Interestingly, bath application of 50 nM CCL2 induced a calcium rise in both neuronal and non-neuronal cells. In neurons, CCL2 produced a mean calcium rise peak of 142.5 ± 87.8% (n = 5, Figure 6A) whereas in non-neuronal cells the calcium response reached 57.8 ± 42.3% (n = 4, Figure 6B). Pre-incubation of the spinal cord slices in AZ889 (100 nM) before (5 minutes), during and after the application of rCCL2 (50 nM) significantly blocked the calcium response in both neuronal and non-neuronal cells (n = 4, Figure 6A-B).

## Discussion

Here we provided integrated cellular and pharmacological evidence revealing the involvement of CCR2 in the enhancement of neuronal and non-neuronal excitability of sensory and CNS cells after nerve injury. Furthermore, our data indicated that these changes in cellular excitability could be an underlying mechanism of neuropathic pain since both cellular activity and pain scores were attenuated by a selective CCR2 antagonist, AZ889. Finally, we identified that analgesia produced by CCR2 antagonism had a major site of action in the spinal cord where it interfered with pathophysiological sensitization revealed by evoked wind up. Overall we provided further evidence to support CCR2 antagonists as an attractive therapeutic approach for neuropathic pain.

Various lines of evidence have identified CCR2 as an important mediator of neuropathic pain. CCR2 expression was increased in various human inflammatory conditions [29] and in rodent neuropathic models [2,11,19,20,30]. Functionally, CCL2 caused cellular excitation modifying information processing [2,4,14,18-20,24]. Mice with CCR2 gene deletion did not develop mechanical allodynia following nerve injury [2] which strengthened the hypothesis that CCR2 signalling could be required for the development of pain hypersensitivity. In addition, CCL2 injections were shown to decrease pain thresholds indicating that CCR2-CCL2 signalling was also sufficient to develop a hyperalgesic state [18,31]. Other studies have identified pharmacological interventions that prevent hyperalgesia development if CCL2 is neutralized prior to the neuropathic induction [12] giving insight into an eventual CCR2-mediated treatment for neuropathic pain. CCR2 antagonists have been shown to attenuate neuropathic pain [4,15] but, although well characterized, their selectivity against other targets and pharmacokinetic properties remain unknown.

In our study, a novel selective pharmacological tool, AZ889, was used to characterize the CCL2/CCR2 effects on pain processing cells and to show that these effects, particularly in the spinal cord, could account for AZ889-mediated antinociception. The differentiation with previously characterized CCR2 antagonists is based on the particular properties of the compound but also on the integration of *in vitro *and *in vivo *characterization to understand the site of action by comparing different routes of administration and using exposure measurements in relevant tissue to confirm effects. The selectivity of AZ889 was used to confirm that the reversal of heat and mechanical hyperalgesia is caused by a selective block of the CCR2 receptor. For this reason, the selectivity profile of AZ889 was assessed in detail against a panel of over 70 targets and revealed IC50 values greater than 10 μM at each of them. The calcium mobilization assay demonstrated that AZ889 is 60 times more selective for CCR2 over CCR5 in blocking the calcium response mediated by these receptors. *In vitro *effects of CCL2, which preferentially binds to CCR2 receptor [21,32], were blocked by AZ889 suggesting a specific CCR2 excitatory mechanism. Exposure values of AZ889 required to obtain antinociception are less than 5 fold of the *in vitro *CCR2 IC50 and within the 60 fold selectivity window against other targets suggesting that pharmacologically relevant doses are sufficient to produce a behavioural effect. Thus, AZ889 represents a novel, potent, selective and functionally active antagonist for the CCR2 chemokine receptor producing analgesia *in vivo *in the CCI model at concentrations that are relevant *in vitro*.

Our results demonstrated novel mechanisms activated by CCR2 that induced intracellular PLC-dependent calcium mobilization and a modulation of membrane excitability that was translated into increased action potential output from DRG neurons. CCL2-evoked activation was also observed as a calcium-mediated excitation occurring in engineered cells expressing CCR2 as well as in both neuronal and non-neuronal cells from the DRG and in the spinal cord. Additionally, we provided pharmacological verification that CCL2-evoked effects were mediated by CCR2 by using the selective antagonist AZ889 which confirmed the importance of this receptor for cellular activation in neuropathic conditions.

Reports analyzing CCR2 expression in neuropathic conditions showed either predominantly non-neuronal CCR2 [14,30] or presence of the receptor in both neuronal and non-neuronal cells [18-20]. It is however possible that different neuropathic models used could explain variations in CCR2 expression. In the present study, functional assays were used to show that CCL2 activated both neuronal and non-neuronal cells in the spinal cord and DRG. Despite this observation, our results could indicate either the presence of CCR2 on both cell types or that CCL2-evoked neuronal activation is indirectly mediated following glial CCR2 excitation and release of a gliotransmitter onto neurons. In the later case, perhaps glial CCR2 could be involved in both CCR2-mediated hyperalgesia and in the analgesic effect produced by AZ889.

CCL2-evoked activation of DRG cells had been previously reported [24]. To our knowledge, our results were the first demonstrating CCL2-evoked excitation of both neuronal and non-neuronal cells in the spinal cord. This excitatory effect appeared to be mediated by CCR2 since it was prevented by the CCR2 antagonist AZ889. Our results indicating the sensitivity of spinal cord cells to CCR2 signalling confirmed the cellular basis of the spinal site of action of AZ889-produced analgesia.

Differences in sites of action have a major implication on drug optimization for therapy. The site of action of CCR2 antagonists producing analgesia remains a matter of debate. A peripheral site of action was proposed based on the predominantly peripheral expression and activation of CCR2 in a demyelination injury model of neuropathic pain [17]. CCR2 expression in sensory cells could support either a peripheral or a central site of action of CCR2 in analgesia depending on the particular location of the receptor (nerve, cell body or axon terminals in the spinal cord). A central site of action was suggested based on the spinal CCL2 upregulation and microglia activation in the spinal cord of CCI animals [12,30]. Two other findings confirmed a spinal site of action of analgesia produced by CCR2 antagonists: first, mechanical allodynia was attenuated following intrathecal injection of CCL2 neutralizing antibody [12] and second, release of CCL2 was increased in the spinal cord of CCI animals following supramaximal electrical stimulation of dorsal roots [12].

Overall, our results were compatible with a primarily central site of action of analgesia produced by AZ889. First, pharmacokinetic studies in vivo demonstrated that AZ889 crossed the blood-brain barrier after systemic administration. Second, intrathecal or systemic dosing of AZ889 in CCI neuropathic rats produced similar anti-nociceptive efficacy in the thermal hyperalgesia behavioural assay suggesting that analgesia was generated in the spinal cord at concentrations of AZ889 confirmed with exposure measures fundamental to ensure efficacy and selectivity. Third, the predominant spinal site of action of CCR2-based analgesia was also confirmed when local application of AZ889 to the spinal cord inhibited WDR neuron excitability. Finally, we identified that AZ889 prevented spinal wind-up of C-afferent fibre response suggesting, for the first time, that interference with synaptic plasticity in the spinal cord could be the mechanism underlying the analgesic properties of this compound and CCR2 antagonists in general. The discrepancy in site of action between studies could be attributed to the differences in animal models of neuropathic pain used and dependent on the particular pathophysiology of nerve injury.

The analgesic profile of AZ889 was also compared with the gold standard benchmark drug pregabalin [33,34]. As previously reported we showed antinociception produced by pregabalin [35,36] with limited efficacy to reverse thermal hyperalgesia in the CCI model and a noticeable decrease in muscle tone at the highest dose. AZ889 demonstrated limited efficacy for heat hyperalgesia as well, but the effect was obtained with a dose 10-fold lower than pregabalin without noticeable side effects suggesting CCR2-based analgesics could have comparable efficacy to available drugs in the clinic while providing increased safety. The ability of AZ889 to reverse completely mechanical but not heat hyperalgesia could also indicate that a treatment for neuropathic pain based on a CCR2 antagonist may be specific for particular indications or modalities. Finally, although blocking CCR2 signalling had been shown to prevent the development of neuropathic pain, here we showed that the more clinically relevant CCR2 antagonism could reverse an established hyperalgesia state in a neuropathic model. Overall, these studies indicated that CCR2 antagonism had the ability to inhibit and reverse both pain behaviour and its neuronal correlates.

## Conclusions

We have revealed important contributions of CCR2 receptors on spinal neurons and on evoked excitability in the spinal cord related to neuropathic pain. We believe behavioural analgesia shown at relevant pharmacological exposures of AZ889 is mediated by major actions via the spinal cord and that these data support therapeutic interventions in neuropathic pain by attenuating central CCR2 systems.

## Methods

### Animals

This study was conducted under a protocol that has been approved by an ethical committee. The animals were kept and experiments were performed at our main site (AZRDM: AstraZeneca R&D Montréal) or at a site which has accreditation from: CCAC (Canadian Council on Animal Care), AAALAC (Association for the Assessment and Accreditation of Laboratory Animal Care) and/or approved by AZ GVC (AstraZeneca Global Veterinary Council) for study conduct. Male Sprague-Dawley rats (Harlan) were group-housed in ventilated cages in a controlled environment room (12-hour light/dark cycle, 20.5-23.5 °C, relative humidity: 40-70%) with food (14% Protein Rodent Maintenance Diet, Harlan Teklad) and water *ad libidum*.

### Drugs

AZ889, a piperazinecarboxamide derivative, was synthesized at AstraZeneca R&D Alderley Park (UK), prepared according to the experimental procedure described in the patent application WO-2006/067401 [37] and selected over other compounds based on its properties in terms of *in vitro *potency, bioavailability, half-life and CNS penetration. All culture reagents were purchased from Wisent or Sigma unless specified. rCCL2 and mCCL2/JE/MCP-1 were purchased from R&D System. Pregabalin was purchased from 3B Scientific Corporation.

### Calcium mobilization in heterologous system

Briefly, HEK293 s cells stably expressing rat CCR2 and the chimeric Gαqi5 were plated (15000 cells per well) in 384-well assay plates in Dulbecco's modified Eagle's medium (DMEM) for 24 h in a humidified atmosphere at 37°C in 5% CO_2_. After removing the medium by inversion, cells were loaded with the calcium-sensitive dye Fluo4-AM (Molecular Devices), 15 μL per well of 4 μM FLUO-4/Pluronic acid mix (1 mM) in buffer and incubated at 37°C for 1 h in a humidified chamber (5% CO2/95% air). Cells were washed 4 times in Hanks' balanced salt solution buffer supplemented with 20 mM HEPES + 0.25% bovine serum albumin at pH 7.4. Different concentrations of the test compound (from 10 mM stock in dimethyl sulfoxide) were added in buffer for 30 min at 37°C in 5% CO2. In this assay, mouse CCL2 was added at 1 nM in buffer (EC50 value in Figure 1) and mobilization of intracellular calcium was measured online (FLIPR, Molecular Devices). The EC50 and IC50 values were determined using 8-point concentration-response curve using non-linear regression and a four-parameter logistic model (Prism 4.03 software, GraphPad Software Inc).

### Model of peripheral nerve injury

Chronic nerve constriction injury (CCI) was induced as described previously [38] in male Sprague-Dawley rats weighing 125-150 g at the time of surgery. Briefly, under isoflurane anaesthesia, a small incision was made 0.5 cm below the pelvis, and the *biceps femoris *and the *gluteus superficialis *(left side) were separated. The sciatic nerve was exposed, isolated, and four loose ligatures (4-0 chromic gut) with 1 mm spacing were placed around it. The nerve was placed back in its natural position and the incision sealed with tissue adhesive (VetbondTM). The animals were allowed to recover for 7-10 days before conducting any behavioural testing. Animals that were not gaining weight or were showing clinical signs of distress (e.g. paralysis, self-mutilation, extensive alopecia) were not included in this study.

### Preparation of DRG cultures

Ganglia from L4 to L6 dorsal roots were harvested from CCI animals, 4 to 19 days post-surgery. The tissue was enzymatically digested at 37°C for 90 min with collagenase-A (2 mg/mL) and papain (1 mg/mL) in DMEM. Cells were plated on Poly-D-Lysine coated coverslips and maintained in F12 supplemented with 10% heat inactivated horse serum, 2 mM glutamine-penicillin-streptomycin, and 2 ng/ml NGF and GDNF for 1-3 days in a humidified chamber (5% CO2/95% O2)_._

### Preparation of spinal cord slices

Spinal cord slices from L4 to L6 region were prepared from CCI animals, 4 to 19 days post-surgery, following guidelines by Wan and colleagues [39]. In order to prevent excess bleeding, 20 uL of Xylocaine was injected intramuscularly at several sites on both sides of the spinal cord. Under deep anaesthesia a laminectomy (T11 to L5) was performed. Once exposed, the spinal cord was isolated and submerged in oxygenated ice-cold solution containing (in mM): 250 sucrose, 5 KCl, 2 MgSO4, 1.25 NaH2PO4, 26 NaHCO3 and 10 glucose. The membranes were carefully removed under the microscope. The tissue was placed in a purposely-built agar block with a conical hole to support the spinal cord in an upright position to cut the transverse slices (300 μm) which were transferred to room temperature ACSF for calcium imaging containing (in mM): 117 NaCl, 4 KCl, 2.5 CaCl2, 1 MgCl2, 1 NaH2PO4, 25 NaHCO3 and 11 glucose.

### Whole cell patch clamp recordings

DRG cells maintained for 1-3 days in culture were transferred to a recording chamber fixed to the stage of an inverted microscope (Olympus) and constantly perfused (0.5 mL/min) with Dulbecco's phosphate buffered saline (PBS), a standard extracellular bathing solution containing (in mM): 137 NaCl; 8.1 Na_2_HPO_4_•7H_2_O; 2.68 KCl; 1.47 KH_2_PO_4_; 0.9 CaCl_2_; 0.5 MgCl_2_•6H_2_O at pH 7 and 275 to 290 mOsm. Whole-cell current-clamp recordings were performed using an Axopatch 200B amplifier (Molecular Devices). Signals were filtered at 1 kHz, digitized at 10 kHz (Digidata, Molecular Devices) and captured and analyzed off-line (Pclamp9 software, Molecular Devices). Action potentials were recorded with borosilicate glass patch pipettes (2-6 MΩ) filled with solution containing (in mM): 110 K-gluconate, 20 KCl, 10 EGTA, 8 NaCl, 10 HEPES, 1 MgCl2 and 4 Mg-ATP at pH 7.3. Action potentials were evoked through injection of depolarizing currents and averaged firing frequency (3 consecutive current steps) was normalized to the baseline frequency: % of evoked action potentials = (evoked frequency of firing (Hz) by the drug/evoked frequency of firing during the baseline (Hz)) × 100. CCL2 was applied within 50-100 μm from the cell body (DAD system, ALA Scientific). rCCL2 was dissolved at 1 μM in PBS containing 0.01% bovine serum albumin and diluted to final concentration in PBS. CCR2 antagonist AZ889, dissolved in dimethyl sulfoxide and diluted in PBS to its final concentration, was applied through the bath perfusion system. Capsaicin 1 mM stock solution was dissolved in dimethyl sulfoxide and diluted to its final concentration in PBS.

### Calcium imaging recordings

Cells and spinal cord slices were loaded with Fluo4-AM (4 μM, 30 min) in an oxygenated chamber (5% CO2/95% O2). DRG cells and spinal cord slices were washed in PBS and ACSF respectively and placed in a chamber mounted on the stage of a fluorescent microscope (Olympus). Tissue was submerged and perfused continuously with PBS (DRG cells) or oxygenated ACSF (spinal cord slices). For each time-lapse image, the fluorescence intensity (F) was averaged over the soma area. Changes in fluorescence (ΔF) were measured as relative changes from baseline fluorescence (F_0_) and expressed as:

$$\%\Delta {\text{F/F}}_{\text{0}}{\text{=[(F-F}}_{\text{0}}{\text{)/F}}_{\text{0}}\text{]}\times 100$$

Calcium responses were elicited by local application of CCL2 and AZ889 was applied in the bath through the perfusion system.

### Behavioural measurements of nociception

Animals were brought to the laboratory 24 h prior to behavioural testing for acclimatization. To measure thermal hyperalgesia [40] animals were placed individually, and allowed to acclimatize for 30 min, on the glass surface (maintained at 30°C) of the thermal stimulator apparatus (IITC Life Science). A radiant heat beam was focused onto the plantar paw surface of the injected limb. The thermal nociceptive response was defined as the latency between onset of stimulation and paw withdrawal. The stimulus intensity was set such that withdrawal latency of naïve rats was approximately 11 s and stimulus duration was limited to 20 s to prevent tissue damage in the absence of a withdrawal response. Baseline thermal testing was performed to identify and removed from the study animals that did not develop hyperalgesia (approximately 20% of animals), e.g. withdrawal latency greater than 8 s and insufficient to distinguish from naïve. Paw withdrawal latencies were calculated as the mean of two trials conducted with 5 min intervals between measures.

Mechanical hyperalgesia was assessed using the analgesia meter (Ugo Basile). Animals were gently restrained, and a steadily increasing pressure was applied to the dorsal surface of a hind paw via a probe with a dome-shaped tip (diameter of 1 mm). The pressure required to elicit paw withdrawal was determined with cut off set at 295 g to prevent tissue damage.

CCI rats and their corresponding naïve controls were used throughout this study. Our historical data showed sham-operated animals do not behave differently than naïve animals and thus were not used for this particular study. Testing occurred at postoperative day 14. In order to assess behavioural pathology, baseline hyperalgesia was determined 1-2 days before pharmacological testing. Animals were then randomized and allocated to treatment groups. Group sizes were based on the need for achieving a minimum statistical power of 80%. In all cases the experimenter was blind to the treatments received. Data were collected from experiments performed by different scientists to avoid any experimental bias. For systemic dosing, AZ889 was dissolved in 0.5% methylcellulose plus 0.1% Tween 80 in distilled water and delivered p.o. For intrathecal administration, injections were performed under isoflurane anaesthesia and the rats were placed on a rounded surface to create an outward curvature of the spine (humped back). By palpation of the spine, the L5-L6 vertebrae were located and a 25G needle, connected to a microsyringe, was inserted perpendicular to the vertebral column between L5-L6 vertebrae. AZ889 (1-30 nmol) or its vehicle (saline) were injected in a volume of 15 μL/rat and tested 15 min later when the animals had completely recovered from the light anaesthesia. The percentage of anti-hyperalgesia for each rat was calculated according to the following equation: % anti-hyperalgesia = [(value-vehicle)/(naïve-vehicle)]*100. The highest dose of AZ889 was also tested subcutaneously (30 nmol/15 μL/rat) to rule out peripheral contribution. Sedation and other locomotor activity side effects were monitored with a TruScan photo beam tracking device (CBI). AZ889 did not induce changes in total movement, rest time, ambulatory distance, vertical entries and stereotypic movements of the rats.

### In vivo electrophysiological recordings

Under isoflurane anaesthesia, the jugular vein, carotid artery and trachea were cannulated. The animals were placed in a stereotaxic frame (Narashige), paralyzed with 2 mg/kg i.v. bolus pancuronium bromide and artificially ventilated (Inspira, Havard Apparatus). Heart rate, ECG, end tidal CO2 and blood pressure were monitored throughout the experimental period and maintained within physiological limits. Core body temperature was maintained at 36.5-37.5°C throughout the experiment by means of a heating blanket connected to a rectal temperature probe. Extracellular single unit signals were digitized for off-line analysis (Spike2 software, Cambridge Electronic Design). Stimulus-evoked responses were analyzed as the total number of spikes recorded per stimulus duration period, and corrected by subtracting the extrapolated spontaneous activity in the same period. Data was normalized for each animal to the baseline evoked-response, calculated as the average of the three values recorded prior to pharmacological administration.

Spinal cord single unit: Extracellular single-unit recordings of deep (500-1000 μm) convergent dorsal horn neurons were made with parylene-coated tungsten microelectrodes (Micro Probes Inc). Electrodes were advanced vertically with a microdrive manipulator through segments L4-L5 of the spinal cord via a laminectomy. The spinal cord was held rigid by clamps rostral and caudal to the exposed section of spinal cord (L4-5) and a small well was formed with the surrounding muscle. Receptive fields of neurons covering one or two ipsilateral toes were identified using brush and pinch stimuli. Responses of neurons to transcutaneous electrical stimuli applied to the centre of the receptive field were recorded. All neurons selected were wide dynamic range neurons (WDR), exhibiting a short-latency Abeta-fibre evoked response (0-20 ms post-stimulus) and Adelta-fibre evoked response (20-90 ms post-stimulus). These neurons also exhibited longer latency C-fibre evoked responses (90-300 ms post-stimulus) and post-discharge responses (300-800 ms post-stimulus). Responses of neurons to brush (artists paint brush) and pinch (calibrated noxious mechanical pinch) stimulation of their peripheral receptive fields were recorded. Three brush strokes and one 5 s pinch stimuli were applied to the receptive field every 10 min and the evoked WDR neuronal activity was characterized. Following identification of a WDR neuron and recording of control, pre-drug brush and pinch-evoked responses (< 15% variance between subsequent control evoked neuronal activity), a 50 μL volume of CCR2 test compound (100 nM or 300 nM) or vehicle (0.1% BSA in saline) was administered directly to the exposed spinal cord [41]. The brush and pinch-evoked responses of spinal neurons were followed for 70 min at 10-min intervals following drug administration.

Nociceptive Flexor Reflex: The activity of alpha-motoneurons was recorded with silver wire electrodes from the nerve to the posterior biceps femoris/semitendinosus muscles in spinalized (T3-T4), anaesthetic free, decerebrated (cranial contents rostral to the mesencephalon) CCI animals [42]. The sural nerve was dissected free and placed on a pair of silver wire stimulating electrodes. The experiment was started one hour after preparation, to allow for recovery from the anaesthetic and spinalization procedures. To quantify the excitability of the flexor reflex activity spikes were counted during the following epochs: (1) 10 s spontaneous activity period; (2) 2 s pinch applied with calibrated forceps (5 × 24 mm rectangular blade surface, sprung at 210 g) on the three middle toes of the ipsilateral hind paw; (3) 12 s supra-threshold electrical stimulation to sural nerve (500 ms square pulse at 1 Hz); each measurement being separated by at least 50 s. Prior to pharmacological administration, baseline values were obtained by repeating the stimulation paradigm for at least 30 min until a stable reflex level was established. Test compound was formulated in 20% cyclodextrin in 0.9% saline (pH 7.2) and infused i.v. to reach a stable plasma concentration. A blood sample was sampled from one test animal to confirm the plasma concentration. The nociceptive responses were measured in a fixed order at 5 min intervals for 60-80 min to obtain maximal drug effects and recovery.

### Determination of AZ889 levels in plasma and brain tissue

A separate group of naïve rats, satellite animals, not subjected to nociceptive testing were used for plasma and brain tissue collection in order to establish the PK/PD relationship of AZ889. At the appropriate time point, satellite animals were injected with drug using the same dosing solution and route of administration as the tested animals. Blood and brain were collected after decapitation. Blood samples were transferred to heparinised tubes and centrifuged at 3000 g for 5 min. Plasma supernatant and brain were then collected and frozen (-80°C).

### Preparation and analysis of plasma samples

An aliquot of 30 μL plasma sample was precipitated by addition of 60 μL of acetonitrile containing 0.1% formic acid and 1 μM of Internal Standard (I.S.). The samples were centrifuged for 30 min at 9000 × g. After centrifugation, a 10 μL aliquot of the supernatant was directly injected for LC/MS/MS analysis. The measurement of AZ889 concentration in plasma was carried out on a generic HPLC coupled to a mass spectrometer with an electrospray ionization source in the positive mode using multiple reaction monitoring. Chromatographic separations were performed using a generic reverse phase HPLC C18 column (2.1 × 50 mm, 3 μ). The mobile phase consisted of 0.1% formic acid in purified water (solvent A) and 100% acetonitrile containing 0.1% formic acid (solvent B). The following gradient was used: 5% to 95% solvent B in 2 min. The flow rate was constant at 0.750 mL/min and the column temperature was set at 45°C. Data processing was performed using the LC/MS/MS software. The first apparent binding constant of AZ889 was determined in 10% plasma by equilibrium dialysis. A total drug concentration of 20 mM was used at a temperature of 37 ± 2°C. The buffer used was isotonic with plasma. The compounds were dialyzed overnight and the resulting samples were analyzed using generic HPLC-UV methodology coupled with Mass spectral peak identification. This value was used to calculate the free fraction of the drug in plasma.

### Preparation and analysis of brain samples

After collection, brains were frozen in liquid nitrogen, pulverized, samples were weighed, and buffer was added (2× the weight). Samples (30 μL) were then homogenized, precipitated by addition of 60 μL of acetonitrile containing 0.1% formic acid and 1 μM of Internal Standard (I.S.) and centrifuged for 30 min at 9000 × g. 10 μL of the supernatant was injected for LC/MS/MS analysis. The procedure for the measurement of AZ889 in brain homogenate is the same as described above for plasma samples.

The degree of brain binding was assessed by equilibrium dialysis utilizing a semi-permeable membrane with a molecular cut-off of 12-14 kDa (HTD Dialysis). The brains of three drug-free control rats were removed and diluted in PBS to prepared tissue homogenate samples. AZ889 (1 μM) was added to the homogenate. In the dialysis unit, 125 μl of samples spiked in homogenate was added to one side of the dialysis chamber and phosphate buffer to the other side. The plate was then placed in a shaker/CO_2_-incubator to equilibrate for 18 hrs at 37°C. Following incubation, 50 μl aliquots from each of the two cells of the equilibrium chamber were collected and transferred to a 96 deep well plate. 50 μl of blank brain homogenate was added to the samples from the buffer side and 50 μl of buffer was added to the homogenate side. An additional 50 μl of water was added to all samples. 150 μl of cold acidified acetonitrile was added to each sample in order to precipitate plasma proteins. Samples were centrifuged and the supernatant analyzed by LC-MS/MS. Diluted brain tissue homogenate were corrected to determine the unbound fraction (Fu) in undiluted tissue:

$${\text{Undiluted Fu = (1/D)/([(1/Fu}}_{\text{2}}\text{)-1]+1/D)}$$

Fu_2 _is unbound fraction measured and D is dilution factor of brain tissue. Control samples were utilized to calculate recovery and stability and the reference compound propanolol was assayed along with AZ889.

### Data analysis

Data for behavioural and electrophysiological studies are presented as a mean of transformed data ± SEM. Unless specified, statistical significance was determined using two-way repeated measures ANOVA followed by a Holm-Sidack post hoc test on normalized data or non-parametric ANOVA followed by appropriate multiple comparison tests (SigmaStat ver. 3.11, Systat Software Inc.). *p*-values of less than 0.05 were regarded as significant. Plasma and brain EC50 values in behavioural studies were determined from 5-point concentration-response curves using non-linear regression and a sigmoidal variable slope logistic model (Prism 4.03, GraphPad Software Inc.). The EC 50 values were defined as the 50% reversal concentration from the regression curve.

## Competing interests

Authors are employed by AstraZeneca R&D Montreal.

## Authors' contributions

AS performed calcium imaging and drafted the manuscript. MP performed in vivo electrophysiology and drafted the manuscript. FM participated in the design of the study and helped draft the manuscript. SJRE performed in vivo electrophysiology and helped draft the manuscript. JM performed behaviour experiments. CJ performed patch clam experiments. EL performed DMPK analysis. PMCL and FV performed in vitro screening. MNP participated in the design of the study and helped draft the manuscript. CQC participated in the design of the study and helped draft the manuscript. All authors read and approved the final manuscript.

## Supplementary Material

Additional file 1 **Mouse and rat CCL2 induce intracellular calcium mobilization in HEK293 s cells expressing CCR2 with similar efficacy**. Mouse and rat derivative of CCL2 evoked calcium activation of HEK293 s cells expressing CCR2 with an EC50 value of 0.5 and 0.8 nM respectively. Calcium mobilization was recorded on a FLIPR system. The data shown is the average of 3 independent experiments. Click here for file

Additional file 2 **In-vitro characterization of AZ889 selectivity against other biological targets**. Selectivity of AZ889 assessed against a broad panel of targets, including (G protein-coupled receptors) GPCRs, ligand-gated receptors, ion channels, transporters and enzymes expressed in a heterologous system. AZ889 was inactive (less than 50% effect at 10 μM) on all targets. Click here for file
