# Supplementary figures and images for: Blocking spinal CCR2 with AZ889 reversed hyperalgesia in a model of neuropathic pain
Source: Mol Pain. 2010 Dec 10;6:90. doi: 10.1186/1744-8069-6-90 (PMC3009975; doi:10.1186/1744-8069-6-90)

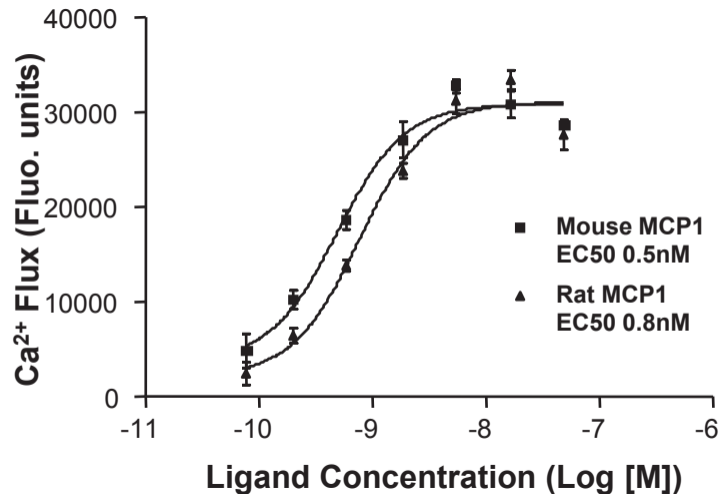

Supplement: Additional file 1 — Mouse and rat CCL2 induce intracellular calcium mobilization in HEK293 s cells expressing CCR2 with similar efficacy. Mouse and rat derivative of CCL2 evoked calcium activation of HEK293 s cells expressing CCR2 with an EC50 value of 0.5 and 0.8 nM respectively. Calcium mobilization was recorded on a FLIPR system. The data shown is the average of 3 independent experiments. [file 1744-8069-6-90-S1.PDF]
