# Supplementary material for: Blocking spinal CCR2 with AZ889 reversed hyperalgesia in a model of neuropathic pain
Source: Mol Pain. 2010 Dec 10;6:90. doi: 10.1186/1744-8069-6-90 (PMC3009975; doi:10.1186/1744-8069-6-90)
Supplement: Additional file 2 — In-vitro characterization of AZ889 selectivity against other biological targets. Selectivity of AZ889 assessed against a broad panel of targets, including (G protein-coupled receptors) GPCRs, ligand-gated receptors, ion channels, transporters and enzymes expressed in a heterologous system. AZ889 was inactive (less than 50% effect at 10 μM) on all targets. [file 1744-8069-6-90-S2.DOC]

**Additional file 2 Table S1**

## In-vitro characterization of AZ889 selectivity against other biological targets.

| **Target / assay** | **% mean inh. at 10µM** |
| --- | --- |
| Acetylcholinesterase Human, Acetylthiocholine Catalysis Spectrophotometry | 2.6 |
| Adenosine Receptor A1 Human, [3H]DPCPX Binding | 11.2 |
| Adenosine Receptor A2a Human, [3H]CGS-21680 Binding | 19.0 |
| Adrenergic Receptor Alpha 1A Rat, [3H]Prazosin Binding | -1.9 |
| Adrenergic Receptor Alpha 1D Human, [3H]Prazosin Binding | 17.4 |
| Adrenergic Receptor Alpha 2A Human, [3H]MK-912 Binding | -5.9 |
| Adrenergic Receptor Beta 1 Human, [125I]Cyanopindolol Binding | -0.4 |
| Androgen Receptor Rat, [3H]Mibolerone Binding | 6.3 |
| Angiotensin Converting Enzyme Rabbit Undefined, FAPGG Catalysis Spectrophotometry | -14.5 |
| Angiotensin II Receptor AT1 Human, [125I](Sar1, Ile8)-Angiotensin II Binding | 1.6 |
| Bradykinin Receptor 1 Human, [3H](Des-Arg10)-Kallidin Binding | 4.2 |
| Bradykinin Receptor 2 Human, [3H]Bradykinin Binding | 13.0 |
| Calcium channel, voltage-dependent, L type, alpha 1 Rat, [3H]Nitrendipine Binding | 14.2 |
| Calcium channel, voltage-dependent, L type, alpha 1 Rat, [3H]Diltiazem Binding | 35.6 |
| Carbonic Anhydrase Human, CO2 staturated H2O Catalysis | 7.9 |
| Cathepsin B Human, Boc-Leu-Arg-Arg-AMC Catalysis Spectrophotometry | -4.5 |
| Cholecystokinin B Receptor Human, [125I]CCK-8 Binding | -6.1 |
| Chymotrypsin Human, Suc-Ala-Ala-Pro-Phe-AMC Catalysis Spectrophotometry | -2.8 |
| CXCR2 Human, CXCL8 Binding | -32.4 |
| CXCR3 Human, CXCL10 FLIPR | -15.0 |
| Dopamine Receptor D1 Human, [3H]SCH-23390 Binding | 1.8 |
| Dopamine Receptor D2 Human, [3H]Spiperone Binding | 8.8 |
| Dopamine Transporter Human, [125I]RTI-55 Binding | 8.5 |
| Endothelin Receptor A Human, [125I]Endothelin-1 Binding | 2.8 |
| eNOS Bovine, [3H]L-Arginine Catalysis Radiometric - HPLC | 13.1 |
| eNOS Bovine, [3H]L-Arginine Catalysis Radiometric - HPLC | 1.9 |
| GABA Receptor Rat, [3H]Flunitrazepam Binding | 5.4 |
| GABAB Receptor Human, [3H]CGP-54626 Binding B1b | -19.2 |
| GABAB Receptor Human, [3H]CGP-54626 Binding B1a | 4.0 |
| Histamine Receptor H1 Human, [3H]Pyrilamine Binding | 0.3 |
| Histamine Receptor H2 Human, [125I]Aminopotentidine Binding | 5.2 |
| Imidazoline I2 Rat, [3H]Idazoxan Binding | -7.9 |
| Insulin Receptor Human, Histone H1 Catalysis Densitometry | 5.0 |
| Leukotriene B4 Receptor Human, [3H]Leukotriene B4 Binding | -8.6 |
| Leukotriene Receptor CysT1 Human, [3H]Leukotriene D4 Binding | -3.0 |
| Matrix Metalloproteinase 2 Human, Mca-Pro-Leu-Gly-Leu-Dpa-Ala-Arg-NH2 Catalysis Spectrophotometry | 8.4 |
| Monoamine Oxidase A Human, Kynuramine Catalysis | 4.4 |
| Motilin Receptor Human, [125I]Motilin (human, porcine) Binding | -0.5 |
| Muscarinic Acetylcholine Receptor 1 Human, [3H]Methscopolamine Binding | 45.9 |
| Muscarinic Acetylcholine Receptor 2 Human, [3H]Methscopolamine Binding | 36.5 |
| Muscarinic Acetylcholine Receptor 3 Human, [3H]Methscopolamine Binding | 9.9 |
| Na+/K+ transporting ATPase Porcine, ATP Catalysis Spectrophotometry | 6.1 |
| Neurokinin Receptor 1 Human, [3H]SR-140333 Binding | 7.5 |
| Neuropeptide Y Receptor 1 Human, [125I]Peptide YY Binding | -6.7 |
| Neurotensin Receptor 1 Human, [125I]Neurotensin Binding | -7.2 |
| Nicotinic Acetylcholine Receptor Human [125I]Epibatidine Binding | 0.7 |
| NMDA Receptor Rat, [3H]CGP-39653 Binding | 25.4 |
| NMDA Receptor-Phencyc Rat, [3H]TCP Binding | 11.4 |
| NMDA Receptor-gly Rat, [3H]MDL105519 Binding | 12.9 |
| nNOS Rat Crude extract [3H]L-Arginine Catalysis(Enzyme Activity) | 8.5 |
| Nociceptin Receptor OPRL1 Human, [3H]Nociceptin Binding | 21.3 |
| Norepinephrine Transporter Human, [125I]RTI-55 Binding | 2.1 |
| Oestrogen Receptor Alpha Human, [3H]Estradiol Binding | -15.5 |
| Opioid Receptor Mu 1 Human, [3H]Diprenorphine Binding | 0.4 |
| Opioid Receptor Delta 1 Human, [3H]Naltrindole Binding | 8.6 |
| Opioid Receptor Kappa 1 Human, [3H]Diprenorphine Binding | 4.2 |
| Phosphodiesterase 3 Human, [3H]cAMP + cAMP Catalysis Radiometric - SPA | -5.2 |
| Phosphodiesterase 4 Human, [3H]cAMP + cAMP Catalysis Radiometric - SPA | 3.8 |
| Platelet Activating Factor Receptor Human, [3H]PAF Binding | 4.5 |
| Prostaglandin Receptor EP4 Human, [3H]Prostagladin E2 (PGE2) Binding | 2.4 |
| Prostaglandin-endoperoxide synthase 2 Human, Arachidonic Acid Catalysis ELISA | 9.7 |
| Prostaglandin-endoperoxide synthase 1 Human, Arachidonic Acid Catalysis ELISA | 20.5 |
| Purine receptor Rabbit, [3H]Alpha, beta-Methylene ATP Binding | 0.6 |
| Purine G Protein-coupled Receptor Rat, [35S]ATP-alphaS Binding | -3.5 |
| 5-Hydroxytryptamine Receptor 1A Human, [3H]8-OH-DPAT Binding | 10.5 |
| 5-Hydroxytryptamine Receptor 2A Human, [3H]Ketanserin Binding | -4.4 |
| 5-Hydroxytryptamine Receptor 3A Human, [3H]GR-65630 Binding | -16.4 |
| 5-Hydroxytryptamine Receptor 4 Guinea Pig, [3H]GR-113808 Binding | 16.9 |
| Serotonin Transporter Human, [3H]Paroxetine Binding | -2.6 |
| Thrombin Human, Z-Gly-Pro-Arg-AMC Catalysis Spectrophotometry | 1.4 |
| Vasoactive intestinal peptide Receptor 1 Human, [125I]VIP Binding | 15.0 |
